# Effects of Online Health Promotion Program to Improve the Health and Wellbeing of Healthcare Students: A Feasibility Study

**DOI:** 10.3390/healthcare12060682

**Published:** 2024-03-18

**Authors:** Maria Shuk Yu Hung, Winnie Wing Man Ng, Edward Kwok Yiu Choi

**Affiliations:** 1 School of Health Sciences, Saint Francis University, Hong Kong, China; 2 School of Nursing, Faculty of Health and Social Sciences, The Hong Kong Polytechnic University, Hong Kong, China; winnie.wm.ng@polyu.edu.hk; 3 Chinese Language Education and Assessment Centre, Lingnan University, Hong Kong, China; edwardchoi@ln.edu.hk

**Keywords:** healthcare students, health promotion, mental health, wellbeing, feasibility

## Abstract

Background: Healthcare students’ health and wellbeing have been seriously affected worldwide. Research studies highlighted the need to establish health promotion strategies to improve them. Methods: A mixed method feasibility with a randomized controlled trial study followed by qualitative focus-group interviews to evaluate the effect of a 24 h online health promotion program improving healthcare students’ health and wellbeing was performed in mid-2022. The study also illustrated the program’s effect, contents, and activity arrangements. Healthcare students from two large tertiary institutions were randomly assigned to intervention and waitlist-control groups. Outcomes were measured by self-completed online questionnaires at three-time points (baseline, week four, and week eight), and in-depth focus-group interviews followed. Results: Among 70 enrolled and 60 eligible students, 54 completed the study, with a 10% attrition rate. Results demonstrated a significant difference between groups at week eight. Within the intervention group, there were significant differences were found from baseline to week eight for depression (*p* = 0.001), anxiety (*p* = 0.004), and stress (*p* < 0.001). The program also improved certain domains of personal wellbeing and quality of life. Qualitative findings further illustrated the program contents and activities’ feasibility, acceptability, and suitability. Most participants welcomed the online mode’s flexibility and convenience. They enjoyed diversified and complementary content and activities. They had increased self-awareness of health and wellbeing. Besides, mental health knowledge enables them to ‘self-care’ and help those in need in the future. Conclusions: The results indicate the feasibility of performing full-scale research in the future and may provide more support for the students of higher education institutions.

## 1. Introduction

“Health is a state of complete physical, mental and social wellbeing and not merely the absence of disease or infirmity” [1] that embraces physical, psychological, and social perspectives of personal health with components of life satisfaction and wellbeing [2]. A significant challenge or adversity may disrupt the homeostatic system and subjective wellbeing (SWB) [2]. Subjective wellbeing, related to satisfaction with life or the quality of life (QOL), can predict mental health status and enhance general health [2]. In the past few years, especially during the COVID-19 pandemic, different populations, including university students, suffering from mental health problems and wellbeing crises have increased universally and locally [3,4,5,6,7,8,9,10,11].

The literature reports that university students’ QOL was negatively correlated with stress. Other influences, such as sleeping problems, depression, and burnout, can further amplify this negative association [12]. However, students in healthcare programs have been more predisposed to mental health problems due to heavy academic study workloads and stressful clinical practicum [12,13,14]. The hectic clinical training in hospitals initiated additional undesirable psychological and physiological influences on their wellbeing due to the pandemic [15,16,17,18,19,20,21,22,23,24]. Mulyadi et al.’s (2021) systematic review and meta-analysis found a high prevalence of health problems among 13,247 nursing students in 17 studies, including sleep disturbances (27%), stress (30%), anxiety (32%), fear (41%), and depression (52%) [19]. Jia et al. (2022) reviewed and analyzed 41 studies of 36,608 medical students and reported that the pooled prevalence of anxiety (33.7%) and depression (37.9%) was comparatively more significant than those of healthcare professionals and the general public [24]. In line with a local study, healthcare students, including medical laboratory science, optometry, nursing, and radiography, had high adverse emotional states [13]. Besides, this study’s authors also investigated 380 local undergraduate general nursing students who reported high levels of stress (39.5%), anxiety (47.6%), and depression (57.1%) [23]. Those who had clinical practicum in hospitals during the COVID-19 pandemic experienced substantial psychological and QOL impacts. The students’ mean scores of all four QOL domains (physical, psychological, social, and environmental) significantly lowered compared to WHO reference data [25] and local frontline nurses [26]. These studies highlighted the significance of early managing students’ health problems and establishing health promotion programs to improve their physical, psychosocial, and mental health and wellbeing [13,17,19,20,23]. Besides, Worsley and her team (2022) analyzed 27 various interventional review research studies conducted over the past two decades to support college and university students’ mental health and wellbeing [27]. The study reported that psychological and interventions conducted through technology were more effective than other means. Most of these interventions focused on enhancing students’ personal mental health. 

Research studies demonstrated laughter therapy’s benefits on QOL, wellbeing, health, and personal development in recent years [28,29,30]. Laughter yoga, a form of laughter therapy, integrates Pranayama yoga breathing and unconditional laughter, developed for years to promote health and wellbeing [31]. Laughter yoga, a positive psychological intervention [32], can relieve mental stress, improve the physiological immune system, and maintain a positive mind during challenging life events [31]. It is a fun, simple, cost-effective, complementary or core therapy suitable for populations with healthy or different medical conditions [29,30]. Research evidence revealed that laughter yoga positively impacted subjective wellbeing [28], lessened clinical nurses’ stress and compassion fatigue [31], and job burnout syndrome [32]. 

Laughter yoga has various benefits through the physiological changes induced by the action of laughter to improve mental health [33]. However, it does not provide sufficient attention to mental health knowledge. Unlike other students, as future healthcare professionals, healthcare students have imperative roles in promoting physical and mental health to the public. To meet the students’ own health needs and future professional roles in health promotion, a health promotion program with additional knowledge and understanding of mental health and disorders is preferable for healthcare students. Thus, a multidimensional comprehensive intervention online health promotion program integrating laughter yoga exercises and Mental Health First Aid (MHFA) training was conducted to fill this gap. The added value of MHFA anticipates broadening the healthcare students’ mental health knowledge, recognizing various mental illness conditions, advancing their health and wellbeing, and cultivating their confidence and competency in offering professional help to those in need in the future. 

MHFA is a standardized psychoeducation training that aims to equip the attendees’ knowledge regarding mental health and common mental illnesses and empower the basic first aid skills to support individuals with mental health problems before getting appropriate professional assistance [34,35]. It was first developed in Australia in 2000 [34] and has been widespread in various countries internationally. The program involves learning warning signs and risk factors of common mental disorders, the “ALGEE” action plan, how to provide initial assistance, where to receive professional support, etc. MHFA programs can be conducted face-to-face or online [36]. In Hong Kong, the face-to-face version was introduced in 2004, and the online version was launched in 2021 during the COVID-19 pandemic [37].

Research studies showed that MHFA was effective and beneficial for different populations, such as community citizens, professionals, and healthcare students, to advance mental health knowledge, understand common mental illnesses, and boost their intention and confidence to assist those individuals with mental health problems locally [38,39,40] and worldwide [36,41,42,43,44,45,46,47,48]. Besides, several MHFA studies reported that e-learning programs were effective and beneficial for healthcare students in Australia [41,49] and the United Kingdom [43]. Local studies demonstrated that face-to-face MHFA training had an extended value in improving general nursing students’ self-awareness of personal mental health [39,40]. However, no evidence has yet revealed the effect of the e-learning program in Hong Kong. 

Positive Psychology can be used as a theoretical framework for integrating laughter exercises and MHFA training. This approach centers around promoting wellbeing, leveraging strengths, enhancing resilience, fostering positive relationships, and cultivating meaning and purpose [50]. Positive Psychology emphasizes the promotion of wellbeing and flourishing, which can be achieved by incorporating laughter exercises to enhance positive emotions and MHFA training to support mental wellbeing. The strengths-based approach encourages individuals to identify and utilize their personal strengths during the interventions, leading to a greater sense of competence and engagement. Enhancing resilience is another crucial aspect, as laughter exercises can help reduce stress and develop positive coping mechanisms.

In contrast, MHFA training equips individuals with strategies to support themselves and others during mental health crises. The interventions also emphasize the importance of positive relationships and social connections, fostering social support and a sense of belonging. Lastly, integrating laughter exercises and MHFA training aims to help individuals find meaning and purpose, leading to greater fulfillment. By utilizing Positive Psychology, these integrated interventions offer a comprehensive and positive approach to mental health promotion and support.

The integrated intervention of the online health promotion program is comprehensive and novel. If successful, the trial can be an encouraging first step towards a new approach to improving the mental health of many students through online. Given the needs and benefits mentioned, our study aimed to evaluate the feasibility of an online health promotion program in improving healthcare students’ health and wellbeing. 

The study’s objectives were:

- to evaluate the preliminary effects of the integrated interventions of laughter exercises and MHFA training in promoting the healthcare students’ health and wellbeing;
- to examine the feasibility of a future full-scale randomized controlled trial (RCT) study;
- to illustrate their experiences and feedback on the online health promotion program’s effects, contents, and activities arrangements.

The research questions of this study were:

- Will the integrated interventions of laughter exercise and MHFA training promote the healthcare students’ health and wellbeing?
- What are the experiences and feedback of healthcare students regarding the effects, content, and activity arrangements of the online health promotion program?

## 2. Materials and Methods

### 2.1. Study Design

This feasibility study was a mixed-methods design using a randomized wait-list-control trial with qualitative focus group interviews followed. Feasibility studies aimed to test the appropriateness of research design, intervention used, and results, and whether the study could be extended or modified to full-scale research [51]. It facilitated the elucidation of the significant issues or concerns regarding this study’s practicality, feasibility, acceptability, etc. The mixed-methods approach enabled the research team to have a comprehensive and better understanding of the data collected and the participants’ views [52,53]. 

### 2.2. Participants and Sampling

The participants were local students recruited from two self-financed tertiary institutions in Hong Kong that offered different professional accredited undergraduate programs, with more than 3000 students studying healthcare-related programs. Purposive sampling was employed. The inclusion criteria were students who were ≥18 years old, were studying healthcare-related programs in Hong Kong, could read and understand Chinese and English, and were willing to attend the arranged activities via the computer Internet. The exclusion criteria were students who had taken laughter yoga and MHFA and those with pre-medical conditions who were neither physically nor psychologically fit for the activities. For sample size estimation, a minimum of 25 samples was required per group with a small effect size (0.2), 15% attrition rate, 90% power, and two-sided 5% significance to assess the study objectives in a two-arm trial [54]. Thus, a total of 60 students was proposed, with 30 students in both the intervention and control groups for this study. 

### 2.3. Ethical Considerations

The Research Ethics Committee of Tung Wah College, Hong Kong approved this study (Ref No. REC2021108) on 1 November 2021, and it was registered at the Chinese Clinical Trial Registry (Ref No. ChiCTR2200056275 2022) in early February 2022. This study was conducted according to the guidelines of the Declaration of Helsinki [55]. Before the study, potential participants were given the research purpose and related information for consideration. Their participation was voluntary, and written consent forms were obtained. The data collected were kept confidential, and anonymity was guaranteed. They could refuse to respond to any items in the questionnaire or answer interview questions. They could withdraw any time, which would not affect their academic performance. 

### 2.4. Recruitment and Interventions 

**Figure 1:**
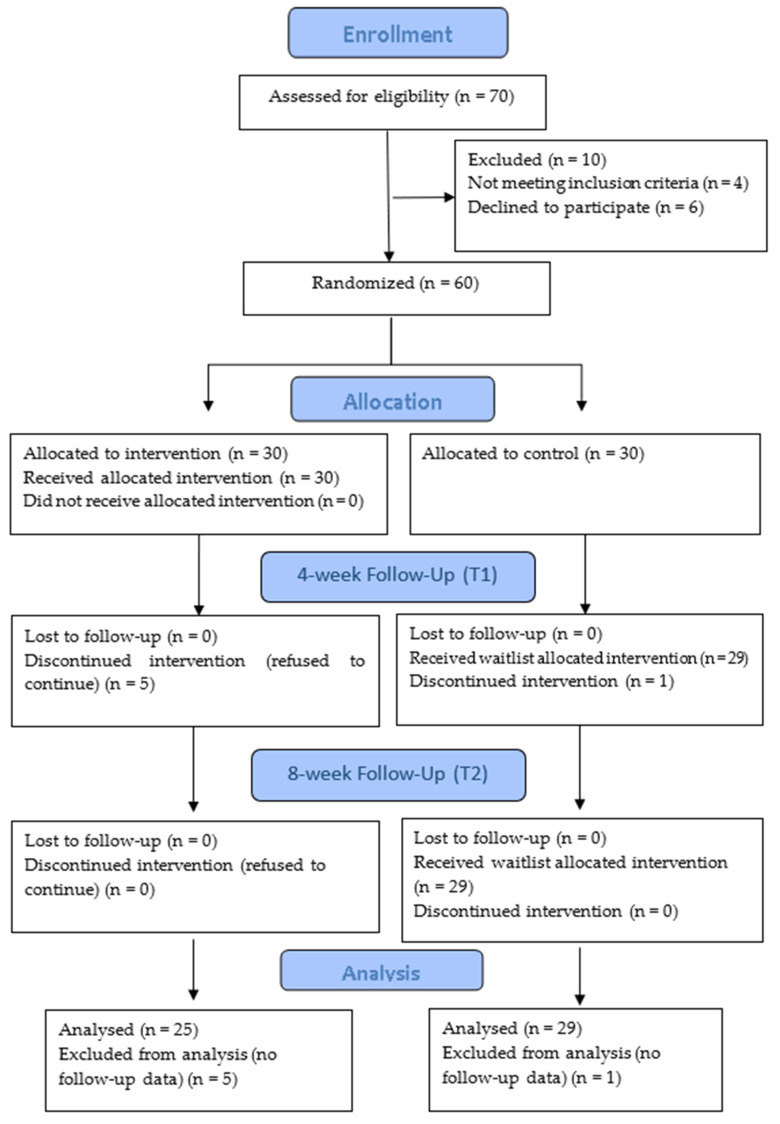
The CONSORT diagram shows the study flow.

The participants’ recruitment commenced in early April 2022. The first author generated a computerized random allocation sequence list, and the students were assigned to the intervention or waitlist control groups accordingly. The Consolidated Standards of Reporting Trials guideline was followed in the study [56], and the study flow is shown in Figure 1. Seventy students studying healthcare programs from two institutions enrolled and were assessed for eligibility. Ten students were excluded. Four did not meet the inclusion criteria (one cannot read Chinese, and three attended a Laugher Yoga leader course), and six declined to participate due to a clash of academic study, clinical practicum, or part-time job schedules. Sixty students were randomized into the intervention group (*n* = 30) and waitlist-control group (*n* = 30) in May 2022. These two groups were separated into two subgroups (two intervention and two waitlist-control) with 15 students/group for better arrangement. 

The health promotion interventions were conducted on weekday evenings for four weeks from late May 2022 onwards. The 12 h laughter activities supplemented with positive psychology exercises comprised 6 h of teacher-led real-time online interactive activities via the Zoom platform, and 6 h of self/small group home practice. The laughter activities were conducted in Cantonese weekly by certified Laughter Yoga Leaders, e.g., clapping hands warm-up exercises, laughter exercises, breathing and relaxation exercises. Short video practice links, such as recordings of real-time teacher-led group [57] self/small group daily practice. Participants were encouraged to integrate laughter and happiness into their everyday lives through daily practice of laughter and breathing exercises. Log sheets were given to record practice and list three grateful things or happy moments daily. Reminders were sent via WhatsApp regularly. The 12 h MHFA training (online version) included 7 h of self-e-learning within the first two weeks and 5 h of teacher-led real-time online sessions conducted by a certified MHFA instructor within the following two weeks. A synopsis of local common mental health problems was introduced, e.g., depression, anxiety, psychosis, substance abuse, suicidal behavior, and panic attacks. Teaching and learning activities include lectures, video demonstrations, small group discussions, and online quizzes. Each participant received the MHFA manual (Hong Kong version IV) and local mental health resources as supplementary information. 

### 2.5. Outcome Measures

Self-completed online questionnaires and in-depth focus group interviews measured the study outcomes. The questionnaires comprised the demographic information and items from different research scales: (1) Depression Anxiety Stress Scales (DASS 21) (Chinese version) [58]; (2) Personal Wellbeing Index—Adult (PWI-A) (Cantonese version) [59]; and (3) WHO Quality of Life Scale Abbreviated Version (WHOQOL-BREF) (Hong Kong Version) [60]. These scales have been well-validated for use in different Chinese populations to assess emotional states, wellbeing, and various QOL domains [57,61,62]. The 21-item DASS [58] included three subscales: Depression (e.g., “I could not seem to experience any positive feeling at all”), Anxiety (e.g., “I was aware of dryness of my mouth”), and Stress (e.g., “I found it hard to wind down”). Items are rated on a four-point scale from 0 (Did not apply to me at all) to 3 (Applied to me very much or most of the time), with higher scores suggesting a severity of mental health problems. The internal consistency in this study was satisfactory: 0.88, 0.84, 0.81 for depression, anxiety, and stress, respectively. The Personal Wellbeing Index (PWI) [59] is evaluated on an 11-point Likert-type scale (0 = no satisfaction at all to 10 = completely satisfied) by 7 questions related to various quality of life domains, including standard of living, personal health, achieving in life, personal relationships, personal safety, community-connectedness, future security, and spirituality/religion. The original scale developer validated the Chinese version. This scale demonstrated acceptable internal consistency in this study with 0.90. The WHOQOL-BREF (Hong Kong version) [60] was used to measure QOL by using a 5-point Likert-type scale (e.g., 1 = Not at all, to 5 = Completely). It consists of 24 items to assess perceptions of quality of life in 4 domains, including physical health, psychological health, social relationships, and environment, and 2 items on overall QOL and general health. The domain scores were transformed into a linear scale between 0 and 100, following the scoring guidelines. The internal consistency in this study was satisfactory: 0.79, 0.81, 0.68, 0.87 for physical health, psychological, social relationships and environment, respectively.

Focus group interviews aimed to identify the views through the participants’ expressions and dialogue during interviews [63,64]. Our study participants were free to form groups, which would facilitate constructive group dynamics beneficial to information sharing [65]. An interview guide with open-ended questions encouraged the participants to express their experiences and views on the program comprehensively. For instance, “What do you think about the operational arrangement of the study activities?”, “What are your opinions about the activities adopted in this online health promotion study?”, “What are your views on learning or practicing these activities?”, “How did these activities influence your health?”. Also, probing questions were followed as required.

### 2.6. Data Collection and Analysis

Data were collected from all participants from May to July 2022 by the online survey platform “LimeSurvey” at baseline before conducting the intervention (T1), after completion of interventions in week 4 (T2), and follow-up in week 8 (T3). After completing the interventions and all quantitative data collection (T1, T2, and T3), healthcare students in the control group received the interventions from late July 2022 onwards to benefit all healthcare students who agreed to participate. The Consolidated Standards of Reporting Trials (CONSORT) diagram (Figure 1) shows the study flow. 

Participants’ sociodemographic characteristics were summarized with descriptive statistics. Continuous data were expressed in Mean ± SD when the data followed a normal distribution, while the Median (25th–75th percentile) was presented if data were not normally distributed. Categorical data were expressed in absolute proportions N (%). An Independent-sample *t*-test was used to compare group differences with continuous sociodemographic variables, which follow the normal distribution. Pearson Chi-square test or Fisher’s Exact test was used to compute group differences with categorical variables. 

About the scales, as the continuous variables violated the normal distribution assumption, a Mann–Whitney U test was applied for between-group comparison. The Wilcoxon signed-rank test was used for within-group analysis. Holm–Bonferroni correction was employed to avoid the inflation of Type I error due to the multiple comparisons [66]. The related samples of categorical variables were analyzed using the Marginal Homogeneity test. Statistical analyses were performed using IBM SPSS Statistics for Windows, Version 26.0 (IBM Corp., Armonk, NY, USA computer software). A *p*-value of <0.05 was considered statistically significant.

After initial survey data analysis, intervention group participants were invited to freely join any of the 4 face-to-face focus-group interviews in late July 2022, with 15 out of 25 participating. Using the semi-structured interview guide with open-ended questions, they were encouraged to express their views about the study arrangement and interventions received. Field notes were recorded during and after the interviews regarding important observations from participants’ body gestures and physical expressions to capture their verbal and non-verbal cues. The interviews were ~40 min on average, were audio-recorded with consent, and transcribed. The field notes were then incorporated into the verbatim transcripts. The first and second authors confirmed the transcripts with the recordings to ensure the correctness of the transcription. After reading and re-reading, the transcripts were analyzed for content. Constant comparison analysis was used to analyze the focus group data collected to disclose, articulate, and elucidate the meaning through three stages [67]. Firstly, the data were separated into smaller pieces with the same central meaning among the healthcare students, with a code attached (open coding). Secondly, the codes were grouped into categories of different meaningful units (axial coding). Finally, themes that illustrate the contents of individual groups (selective coding) were established to identify the healthcare students’ experiences and feedback regarding the health promotion program. 

Trustworthiness is essential to rigorous qualitative research [68]. According to Guba (1981), trustworthiness is achieved through credibility, confirmability, transferability, and dependability [69]. Several techniques were employed to enhance trustworthiness in this study. Credibility was ensured through peer debriefing sessions with the first and second authors, allowing for critical evaluation and identification of flaws. Confirmability was addressed through member-checking with all participants, validating the interpretation of findings [70]. Transferability was facilitated by providing a detailed description of the study’s context, enabling findings to be applied to similar situations [71]. Dependability was achieved through a comprehensive operational description of the research methodology, allowing future researchers to replicate the study. Collectively, these techniques enhanced the trustworthiness of the study.

## 3. Results

### 3.1. Quantitative Results

#### 3.1.1. Baseline Sociodemographic Characteristics and Background

Among 70 enrolled and 60 eligible students who participated, 54 completed the study, with 25 in intervention and 29 in waitlist control groups. The results were promising, with a 90% retention rate (5 and 1 dropout in intervention and waitlist-control groups, respectively). Table 1 shows the sociodemographic characteristics and background of the participants in both groups. The mean age of the participants was 22.11 years, and 79.6% were female. Over 50% of the participants were studying a Bachelor Degree. Approximately 22.2% of the participants attended clinical practicum during COVID-19. All participants agreed that the MHFA course could improve health, and 94.4% agreed that Laughter Yoga could improve health. No statistically significant differences between the two groups regarding the sociodemographic and background were observed.

#### 3.1.2. Ratings of DASS-21, PWI, and WHOQOL-BREF, and between the Intervention and Control Groups

The students were asked to rate their emotional states of depression, anxiety, and stress by using DASS-21 at baseline (T1), week four (T2), and week eight (T3) after the intervention. The intervention group scored significantly lower than the control in the depression subscale at week eight (T3, *p* = 0.023). There was no between-group difference at week four (T2, *p* = 0.149). Within the intervention group, significant decreases were found from baseline (T1) to week eight (T3, *p* = 0.001) and from week four (T2) to week eight (T3, *p* = 0.016). For anxiety, there were no significant group differences at week four (T2) and week eight (T3) in the anxiety subscale between the intervention group and the control group. Within the intervention group, significant decreases were found from baseline (T1) to week four (T2, *p* = 0.01) and from baseline (T1) to week eight (T3, *p* = 0.004). For stress, there were no significant group differences between the intervention group and the control group in the stress subscale at week four (T2) and week eight (T3). Within the intervention group, significant decreases were found from baseline (T1) to week four (T2, *p* = 0.005); from baseline (T1) to week eight (T3, *p* < 0.001); and from week four (T2) to week eight (T3, *p* = 0.035). Table 2 shows the between- and within-group comparisons of DASS 21 over three time points.

The PWI Scale was adopted to assess the subjective wellbeing (SWB) of different domains regarding the healthcare students’ feelings about themselves. Within the intervention group, the score of “Average subjective wellbeing” was significantly higher from baseline (T1) to week four (T2) (*p* = 0.004) and from baseline (T1) to week eight (T3) (*p* = 0.004). Table 3 shows the between- and within-group comparisons of PWI over three time points.

The WHOQOL-BREF was used to examine the four domains for the QOL. Within the intervention group, the score was significantly higher from baseline (T1) to week four (T2) in the Physical Health domain (*p* = 0.024) and Psychological domain (*p* = 0.016); from baseline (T1) to week eight (T3) in the Physical Health domain (*p* = 0.002), Psychological domain (*p* = 0.001) and Social Relationships domain (*p* = 0.003); from week four (T2) to week eight (T3) in the Psychological domain (*p* = 0.007). No significant group differences in week four (T2) and week eight (T3) in all domains. Table 4 shows the between- and within-group comparisons of WHOQOL-BREF (Hong Kong version) over three time points.

### 3.2. Qualitative Results

#### 3.2.1. Baseline Sociodemographic Characteristics and Background

Among the 15 intervention group students who participated, 13 were females. Ten, four, and one student were studying Bachelor, Higher Diploma, and Associate programs, respectively. Their experiences and feedback regarding the online health promotion program’s effects, contents, and arrangements were illustrated in two themes: (1) Increased self-awareness of health and wellbeing, and (2) enjoyed diversified and complementary content and activities, with eight categories. The themes, categories and some codes (examples) identified from the interview data are listed in Table 5. 

#### 3.2.2. Effects on Health and Wellbeing—Increased Self-Awareness of Health and Wellbeing

The first theme, “increased self-awareness of health and wellbeing”, relates to the substantial effects on health and wellbeing, perceived as attribution to experiencing different health benefits through the interventions, including physiological, psychological, social, and attitudinal aspects. Many participants expressed that their awareness of health conditions was enhanced by the activities, knowledge, and skills they learned after joining the study. One participant narrated her experience:

“*The most significant and impressive message I appreciated was ‘to take good self-care before caring for others’. I learned mental health knowledge and could identify the warning signs of different mental disorders. I became more alert and aware of my health and emotions than before. When I was not in a good mood or encountered academic stress, laughter and breathing exercises helped me relax and calm my emotions.*” (Group 4 Participant 3)

##### Physiological Fitness and Health Improvement

Concerning this category, participants experienced various physiological improvements, including relaxing physically and feeling comfortable, improving insomnia and sleeping conditions, and feeling refreshed and energized by practicing laughter activities. One participant conveyed her feelings about the daily practice of laughter activities:

“*Since joining, I have had daily self-practice of laughter, breathing, or relaxation exercises after dinner for about 20 min, feeling relaxed and refreshed after activities. I could start studying again, feel more energetic and effective, lessen stress, improve my insomnia, and sleep better.*” (Group 4 Participant 2)

Another participant further elaborated on her perceived benefits to physical health:

“*Through daily practice of laughter and breathing exercises, I feel my heart becoming robust and my lung capacity increasing. It’s effective in improving my physical health.*” (Group 3 Participant 2)

##### Psychological and Emotional Balance

Another category identified was the importance of achieving and maintaining a psychological and emotional balance during their daily lives. Nearly all the participants expressed that the activities could help relieve their stress and anxiety, convert negative moods, and reinforce happiness and positive minds. One participant mentioned what happened to her mood changes through the laughter activities:

“*Initially, I didn’t understand how to make a false laugh into a true one. But it really happens when practice. I experienced and felt myself relaxed and happier. After doing laughing and breathing exercises, I find myself with decreased anxiety and can concentrate more on my studies without distraction.*” (Group 3 Participant 3)

Another participant echoed this positive benefit of handling emotional stress caused by the academic study:

“*I used to have low EQ (Emotional Intelligence Quotient). I’m easy to get agitated, especially when I am under stress due to my study. After learning the MHFA and the laughter activities, I know more about relaxing and stabilizing my emotions.*” (Group 4 Participant 3)

##### Social Wellbeing and Connection 

The social wellbeing and connection category illustrated the positive effects on social interaction and relationships. Some participants stated that the activities facilitated connections and relationships with others and strengthened their communication skills and self-confidence. A student mentioned connecting and increasing confidence with her classmates using the activities:

“*I practice the activities with my classmates before our project presentations or assessments. The slogan, ‘Very good, Very good, yeah! Hoho, Hahaha!’ It’s upbeat and helpful for self and others’ encouragement. It can boost our mood and confidence.*” (Group 3 Participant 2)

Another participant stated the effects of group playful laughing exercises buffering stressful situations and enhancing relationships:

“*The unconditional laughter and childlike playfulness exercises are so funny and stress relieving. I do it with my friends when we have time, whether stressed or not in a good mood. We like laughing together for joy, better morale, and rapport.*” (Group 1 Participant 1)

##### Attitudinal Change in Health Concepts 

Several participants stated that the activities clarified their health concepts and beliefs. Apart from physical health, they become more aware, objective, and thoughtful about mental health issues, particularly the significance of mental wellbeing and changes in attitudes toward people suffering from mental problems. Besides, they experienced training their body and mind to laugh at will. One participant stated her views regarding the association of emotions and behaviors, highlighting her changes in attitude toward the significance of mental health:

“*If I’m sad, I cry; if I’m happy, I laugh. Though I know emotions could affect behavior and *vice versa*. The laughter exercises embarrassed me initially, and I was too shy to laugh without reason. After daily practice of laughter exercises and writing down grateful and happy events, I enjoy and treasure it. After this study, I really experienced that if you ‘pretend’ to laugh, your mimic laugh will gradually become true, as the laughter yoga leader said. You can easily change your mood to be happy, promoting your mental wellbeing. It’s true.*” (Group 3 Participant 1)

Some participants expressed the activities cultivated positive emotions and also inspired their intention to assist individuals suffering from mental problems, especially after receiving knowledge regarding mental health and disorders. The subtle changes reflected the participants’ enhanced willingness to help others, like their family and peers. One participant said:

“*My friend has anxiety and depression and recently suffered from OCD (Obsessive Compulsive Disorder) also. Her emotions were not so stable. The MHFA clarified my concepts, allowing me to understand her problems better and how to communicate with her appropriately. I will try to offer help or advise her what to do.*” (Group 2 Participant 4)

#### 3.2.3. Feedback on Contents and Activities—Enjoyed Diversified and Complementary Content Activities Arrangement

The second theme, “enjoyed diversified and complementary contents and activities”, relates to the feedback on contents and activities. It comprises four categories: enjoyed funny laughter yoga exercises, appreciated grateful and happy events daily records, structured and informative mental health knowledge, and facilitated flexible participation arrangements. Positive comments, such as diversified, engaging, informative, complementary, and flexible, were mainly received regarding the organized contents and activities. One participant voiced out her views:

“*Um, the contents and activities are diversified, interrelated, and complementary. Laughing yoga exercises remind us of ‘self-care’ for our health. It can strengthen our physical health and maintain emotional balance. Daily records of three grateful events and happy moments can improve life satisfaction. Through MHFA, our awareness and mental health knowledge increased as we recognized more about common mental disorders, not just benefiting ourselves but also can assist our family or friends who have mental health problems.*” (Group 3 Participant 3)

##### Enjoyed in Funny Laughter Yoga Exercise

The laughter exercises emphasized the body-mind connection in achieving wellness for good health. Most participants said they enjoyed the funny and excited laughter exercises, though some initially felt shy. Some participants stated that laughter boosted their positive emotions and self-confidence. One participant shared her experience as follows:

“*I was shy during the first laughter yoga session but could enjoy the following sessions. The different exercises were fun and exciting. I found myself becoming more involved and gradually feeling relaxed. I didn’t mind others’ views; I just did what I liked. The exercises uplifted my mood and improved my self-confidence. It helped me a lot. I would be nervous and anxious about the project presentation, but I am no longer nervous now.*” (Group1 Participant 2)

Another participant reaffirmed the two-way natural link between the mind and body attributed to laughter yoga exercises. The participants were more engaged through the leaders’ innovative exercises and encouraging participation. They enjoyed the activities, and one participant said:

“*The teacher is an experienced and innovative leader. We learned how to be happy through the childlike, playful exercises. She encouraged each of us to design and take the lead in a laughter exercise. It’s so funny and pleasurable. I liked breathing and relaxing exercises also. “Humming” (a type of breathing exercise) is helpful. I have mood swings easily, but I can relax and calm down after practicing it, even when stressed preparing for clinical practicum.*” (Group 2 Participant 3)

##### Appreciated Grateful and Happy Events’ Daily Records

Most participants believed the bring-home activity was helpful, reminding them of positive thinking about those nearly forgettable, grateful events, happy moments, and compliments for everyday life. However, a few participants occasionally forgot daily records or thought written records were unnecessary. They had the opportunity to share their joy during weekly group sessions briefly. For instance, one participant described her thoughts:

“*I enjoy recording happy moments daily, though sometimes I forget. It reminds me of the events I have nearly neglected but can make me happy. The idea of compliments also makes me and others happy. It’s nice!*” (Group 2 Participant 4)

Besides, some participants stated that daily recording made them feel more satisfied with life and an increasing sense of gratitude and appreciation. Another participant further elaborated on her feelings and reflections regarding the records:

“*I like the log sheet. My daily life is filled with study stress and anxiety. I seldom think of happy moments. When thinking about and recording the ‘three daily grateful and happy issues,’ I realize I have some inner changes. Daily appreciation can change my mood and life attitude to be more positive and enthusiastic. It reminds me of life’s meaning and satisfaction with positive thinking.*” (Group 3 Participant 4).

##### Structured and Informative Mental Health Knowledge 

A common view among participants was that the materials provided were structured, informative, and practical about mental health knowledge and skills that enriched their knowledge and strategies for mental problems. The information reinforced their understanding and consolidated what they had learned:

“*We learned different common mental disorders and increased our self-awareness of the warning signs and risk factors. The MHFA strengthened our understanding of identifying and initiating help for those with mental problems and referring professional help. We had opportunities to practice communication using different scenarios. It’s practical.*” (Group 3 Participant 4)

Most participants widely accepted the standardized MHFA online version (Hong Kong version), including 7 h self-e-learning of mental health knowledge before 5 h teacher-led discussion and consolidation.

“*I think the contents were good and adequately presented. Self-e-learning is informative with texts, videos, and quizzes and is easy to follow. The two teacher-led consolidation sessions allowed us to ask questions and clarify our concerns. Local case scenarios help to illustrate different situations. The supplementary local resources with contact details are useful. It was a good online learning experience.*” (Group 4 Participant 2)

##### Facilitated Flexible Participation Arrangements

Most participants appreciated that operational arrangements were flexible, convenient, time-saving, and feasible to suit their busy study or work schedules for participation. They affirmed that the activity schedules, such as the time, duration, and frequency, were arranged appropriately.

“*The study conducted in the summer term, including teacher-led and self-practice activities, is acceptable. The weekly online laughter yoga exercises on weekday evenings with daily self-practice of 20 min were feasible. The two weeks of MHFA self-e-learning could be accessed flexibly, and the following two weekends of teacher-led consolidation online sessions were also convenient.*” (Group 3 Participant 2)

Most participants preferred the convenient online and e-learning mode of delivery. One participant explained her preferences and views:

“*I like online and e-learning modes that are time-saving and convenient. As Hong Kongers are usually busily engaged, reserving a fixed date, time, and venue to attend regular activities is not easy. It may cause inconvenience to somebodies. Nighttime is a good time for relaxing. After showering, I can sleep right away. If we attend laughter exercises outside, we must return home first.*” (Group 3 Participant 3)

However, other participants found the online method embarrassing and decreased interaction. They believed face-to-face mode would be more interactive, interesting, and engaging, especially for laughter exercises. They declared they might consider joining face-to-face classes if the venue is nearby and time is available.

“*I agree that online mode is innovative and time-saving. But I felt a bit goofy and strange, laughing at the monitor. My home was small, and my family was watching around and feeling embarrassed. I believe face-to-face mode would be more interactive and better communication.*” (Group 2 Participant 2)

## 4. Discussion

The current feasibility study successfully evaluated the preliminary effects of the online health promotion program on promoting healthcare students’ health and wellbeing. By integrating laughter activities supplemented with brief positive psychology exercises and the MHFA, this multidimensional comprehensive health promotion intervention created a more effective intervention in improving the healthcare students’ mental health and wellbeing. 

The quantitative results supported that the online health promotion program significantly reduced depression, anxiety, and stress during follow-up in week eight (T3) (Table 2). The participants’ wellbeing improved by the program, as reflected by a significant increase in the overall ‘Average subjective wellbeing’ of PWI in week four (T2) and week eight (T3) (Table 3). Also, the results showed that participants’ QOL was enhanced with a rise in the Psychological, Physical Health, and Social Relationships except in the Environmental domains of WHOQOL BREF in week eight (Table 4).

This study results demonstrated that the healthcare student participants’ baseline (T1) mean scores on the WHOQOL-BREF’s four domains were similar to the authors’ previous study of nursing students [23] but lower than the WHO reference information (physical = 16.2, psychological = 15.0, social = 14.3, environment = 13.5) [25]. Indeed, this study’s health promotion intervention focused more on individuals’ mental, physical, and social health and personal wellbeing and showed positive effects. The effects on the Environmental domain might not be explicit. Moreover, the small and overcrowded average living space per person (13.6 m^2^) in Hong Kong [72] and the persistent enactment (without relaxation) of social distancing policies and measures to fight against the COVID-19 pan-demic by the Hong Kong Special Administrative Region Government [73] during the study period might be the potential factors influencing the effects of the Environmental domains of QOL. 

On the other hand, the qualitative findings illustrated the participants’ positive views regarding the study’s arrangements, contents, and effects of the integrated activities of the online health promotion programs that further supported the program’s feasibility, acceptability, practicality, etc. Everyone can laugh anytime and anywhere, free of charge, individually or with family and friends. Laughter Yoga can synchronize the body and the mind, maintaining a mutual harmony in the two-way connection [74]. Our participants’ narratives supported a positive change in their mental state through body exercises initiated by laughter yoga. They recognized the transition from the beginning of mimic laughter into genuine laughter. The study’s quantitative results and participants’ narratives revealed improvements in reducing stress, depression, and anxiety, increasing positive emotions, developing positive coping, and expanding social relationships, consistent with the previous literature [28,75,76]. Also, our participants valued daily records of three grateful events and happy moments. The practice of appreciation and compliments reminded their spirit of thankfulness towards themselves and others, which could improve their wellbeing. In line with the literature, brief positive psychology exercises have demonstrated enhancement in happiness [77], positive affect [78], and life satisfaction [79]. Through the practice of appreciation, their sense of happiness and that of those around them increased [74].

As an established standardized psychoeducational program, research evidence demonstrated that the MHFA improved attendees’ mental health knowledge, reduced stigma, and promoted confidence and helping intentions for those people with mental health problems [45,46,47]. Similar to the first author’s previous face-to-face MHFA training for nursing students locally, this online study enhanced healthcare students’ mental health knowledge and self-awareness of personal mental wellbeing [39,40]. Apart from these benefits, our participants found that the online MHFA program was structured, informative, and practical. It strengthened their understanding, consolidated what they had learned, and increased their self-awareness of mental health and wellbeing. Besides, mental health knowledge enables them to recognize warning signs of mental health problems and thus could have ‘self-care’ and help those in need in the future.

Our study’s overall attrition rate was 10%, lower than other health promotional studies of college students (>40%) [3,80,81,82]. The main reasons for the six students’ dropping out were clashes with clinical practicum (two students) or busy personal engagements (four students). The low attrition rate might be due to the online flexible approach and healthcare students’ interest in and perceived usefulness of these interventions. The participants expressed increased awareness of “self-care” and were willing to integrate what they had learned into their daily lives. Also, the study activities were mainly conducted in the summer semester, and the study schedules were not as packed as in the standard semesters. 

In recent years, online or internet-based interventional studies have been adopted to promote or improve mental health, including MHFA programs and laughter activities [41,43,49,83,84]. For instance, e-learning MHFA programs were effective and beneficial for healthcare students’ in Australia [41,49] and the United Kingdom [43]. Eraydin and Alpar (2022) reported that online laughter yoga exercises had a statistically significant effect on nursing students’ psychological wellbeing and life satisfaction in Turkey [84]. The online approach was practical and feasible with positive impacts. Although the face-to-face mode enables information sharing and better interaction during activities, the online method provides an easy and convenient approach that caters to cost-effective and flexible arrangements [36]. Integrating a fun and engaging approach to laughter yoga exercises with the more established evidence-based intervention of MHFA, our research team intended to initiate a unique and comprehensive online intervention focusing on students’ physical, mental, and social wellbeing. Most participants appreciated the flexible online mode, and the activity arrangements were feasible and suitable for their busy class/clinical practicum schedule. Some preferred face-to-face classes if time was available or the venue was nearby. Future studies may consider a dual mode to encourage sufficient engagement and better social interaction.

Regarding the intervention’s operational arrangement, four weeks’ activities were arranged in different modes, including teacher-led and self-practice, group, and individual. Most participants in our study found the mode, time, frequency, and duration of the online activities and take-home exercises appropriate and effective, similar to the previous literature. An RCT assessed the effect of 1 h real-time online laughter yoga on nursing students for five weeks via the Zoom platform during the COVID-19 pandemic [84]. The results effectively decreased the participants’ anxiety and enhanced their psychological wellbeing and life satisfaction. Another local study reported that brief laughter yoga intervention with take-home practice could reduce Chinese adults’ mental health risks [85]. Besides, the founder of laughter yoga also emphasized the benefits of daily practice and integration into everyday life [86]. 

Although this study was conducted in Hong Kong in 2022 during the COVID-19 pandemic, the adverse impacts on local students’ health, especially mental health, have not yet been relieved but are still increasing in severity until late 2023 [87]. The post-pandemic suicidal tendency might be related to students’ more considerable challenge after normalizing classes and study [88]. Universities or colleges are urged to provide more mental health strategies or support students in increasing their health awareness and seeking help if necessary.

The study had a few limitations. First, the small sample size of the feasibility study limited to healthcare students from two local self-financed tertiary institutions may not represent the overall student population in other institutions. Due to these factors, the generalizability of the results of the present study was seriously limited. This feasibility study aimed to evaluate the preliminary effects and examine the feasibility of integrated laughter exercise and MHFA training interventions to promote healthcare students’ health and wellbeing in a future full-scale randomized controlled trial (RCT) study. Second, an alternative intervention or placebo control group would be preferred in future large-scale studies instead of a control group with no treatment. Third, though the participants from the two institutions were instructed not to disclose any study information to their peers, more strategies could be used to prevent contamination. However, the strength of this study was the flexibility of the online delivery method, which can facilitate healthcare students’ participation in the research and further minimize the dropout rate. Besides, the results of this feasibility study can be generalized to a broader context. As future healthcare professionals, healthcare students have imperative roles in promoting physical and mental health to the public; they might be more concerned about health and wellbeing than other students. By promoting students’ health and wellbeing, this study could advance toward a healthier and more supportive environment for all students in tertiary institutions. 

For future implications, the results of our feasibility study added value to the practical intervention on improving healthcare students’ mental health and wellbeing. The results are also timely in enhancing healthcare students’ mental wellbeing in a post-pandemic era or future pandemic. Therefore, the results have implications for healthcare professionals, faculty in tertiary institutions, and policymakers to develop appropriate strategies, policies, and interventions for supporting healthcare students during hardship and future adversity. The senior management of the institutions can consider this multidimensional mental health care promotion program as an orientation program for new students or an extra-curricular wellness program for all students.

## 5. Conclusions

The global increase in negative psychological impacts on the health and wellbeing of university healthcare students highlighted the urgency and need for adequate support for the students. This feasibility study supported the idea that healthcare students may benefit from an online health promotion program to improve their health and wellbeing. The results revealed an improvement in students’ emotional health, QOL, and wellbeing. The qualitative findings illustrated the participants’ positive views and experiences regarding the study’s effects, contents, and arrangements of the online health promotion program. This program warrants further examination using a full-scale RCT design to support its effectiveness. As future healthcare professionals, we hope healthcare students have good physical and mental wellbeing, and process professional knowledge and caring attributes leading to helping and supporting others in need.

## Figures and Tables

**Table 1:** Sociodemographic Characteristics and background of the participants in both groups.

Variable	All Participants (*n* = 54)	Intervention Group (*n* = 25)	Control Group (*n* = 29)	*p*-Value
Gender				0.95 ^c^
Male	11 (20.4%)	5 (20%)	6 (20.7%)	
Female	43 (79.6%)	20 (80%)	23 (79.3%)	
Age (Mean ± SD)	22.11 ± 4.74	22.20 ± 5.17	22.03 ± 4.43	0.9 ^t^
Educational level				0.241 ^c^
Associate	10 (18.5%)	5 (20%)	5 (17.2%)	
Higher Diploma	9 (31.0%)	3 (12%)	9 (31%)	
Bachelor	15 (51.7%)	17 (68%)	15 (51.7%)	
Major study				1 ^f^
Health Studies	9 (16.7%)	4 (16%)	5 (17.2%)	
Nursing	45 (83.3%)	21 (84%)	24 (82.8%)	
Year of study				0.693 ^f^
Year 1	21 (38.9%)	9 (36%)	12 (41.4%)	
Year 2	15 (27.8%)	6 (24%)	9 (31%)	
Year 3	8 (14.8%)	4 (16%)	4 (13.8%)	
Year 4	7 (13.0%)	5 (20%)	2 (6.9%)	
Year 5	3 (5.6%)	1 (4%)	2 (6.9%)	
Any clinical internship during COVID-19?				0.109 ^c^
Yes	12 (22.2%)	8 (32%)	4 (13.8%)	
No	42 (77.8%)	17 (68%)	25 (86.2%)	
Can MHFA improve your health?				
Yes	54 (100%)	25 (100%)	29 (100%)	
Can Laughter Yoga exercise improve your health?				1 ^f^
Yes	51 (94.4%)	24 (96%)	27 (93.1%)	
No	3 (5.6%)	1 (4%)	2 (6.9%)	

Continuous variables were analyzed by independent-samples *t*-test (^t^). Categorical variables were analyzed using a Pearson Chi-square test (^c^) or Fisher’s Exact test (^f^).

**Table 2:** Between- and within-group comparisons of DASS 21 over three time points.

	Baseline (T1)	Week 4 (T2)	Week 8 (T3)			
DASS 21	Median (25th–75th Percentile)	Median (25th–75th Percentile)	Median (25th–75th Percentile)	*p* (T1 vs. T2)	*p* (T1 vs. T3)	*p* (T2 vs. T3)
DASS 21-depression						
Intervention group	12.00 (6.00–16.00)	6.00 (2.00–14.00)	4.00 (1.00–8.00)	0.053	* 0.001	* 0.016
Control group	12.00 (6.00–17.00)	10.00 (6.00–14.00)	8.00 (3.00–15.00)	0.075	0.029	0.333
*p* (between-group difference)		0.149	* 0.023			
DASS 21-anxiety						
Intervention group	8.00 (4.00–12.00)	6.00 (3.00–10.00)	4.00 (1.00–9.00)	* 0.01	* 0.004	0.753
Control group	8.00 (4.00–13.00)	6.00 (2.00–14.00)	4.00 (2.00–14.00)	0.157	0.031	0.362
*p* (between-group difference)		0.376	0.446			
DASS 21-stress						
Intervention group	16.00 (10.00–19.00)	10.00 (5.00–18.00)	8.00 (2.00–13.00)	* 0.005	* <0.001	* 0.035
Control group	16.00 (8.00–19.00)	14.00 (6.00–18.00)	14.00 (5.00–18.00)	0.142	0.417	0.828
*p* (between-group difference)		0.378	0.058			

DASS 21—Depression Anxiety Stress Scales (Chinese version). * Significant difference. Analysis was adjusted using the Holm–Bonferroni method. Analysis (within-group comparison) was analyzed using a Signed-rank test. Analysis (between-group comparison) was analyzed using a Mann–Whitney U test.

**Table 3:** Between- and within-group comparisons of PWI over three time points.

	Baseline (T1)	Week 4 (T2)	Week 8 (T3)			
PWI	Median (25th–75th Percentile)	Median (25th–75th Percentile)	Median (25th–75th Percentile)	*p* (T1 vs. T2)	*p* (T1 vs. T3)	*p* (T2 vs. T3)
PWI-Average subjective wellbeing						
Intervention group	55.00 (44.38–61.25)	65.00 (48.13–71.88)	66.25 (50.63–75.63)	* 0.004	* 0.004	0.338
Control group	52.50 (43.75–67.50)	58.75 (44.38–66.25)	55.00 (43.75–66.88)	0.698	0.547	0.346
*p* (between-group difference)		0.092	0.056			

PWI—Personal Wellbeing Index. * Significant difference. Analysis was adjusted using the Holm–Bonferroni method. Analysis (within-group comparison) was analyzed using a Signed-rank test. Analysis (between-group comparison) was analyzed using a Mann–Whitney U test.

**Table 4:** Between- and within-group comparisons of WHOQOL-BREF (Hong Kong version) over three time points.

	Baseline (T1)	Week 4 (T2)	Week 8 (T3)			
WHOQOL-BREF (HK Version)	Median (25th–75th Percentile)	Median (25th–75th Percentile)	Median (25th–75th Percentile)	*p* (T1 vs. T2)	*p* (T1 vs. T3)	*p* (T2 vs. T3)
Physical Health						
Intervention group	13.71 (11.14–15.14)	14.86 (12.29–16.86)	14.86 (13.14–17.14)	* 0.024	* 0.002	0.137
Control group	14.86 (13.14–16.29)	14.29 (12.00–16.00)	14.86 (12.29–16.00)	0.141	0.614	0.612
*p* (between-group difference)		0.519	0.343			
Psychological						
Intervention group	12.50 (10.25–13.50)	14.00 (12.00–14.75)	14.00 (12.50–15.75)	* 0.016	* 0.001	* 0.007
Control group	13.00 (11.00–14.75)	12.00 (11.50–14.50)	13.00 (11.25–14.50)	0.318	0.969	0.242
*p* (between-group difference)		0.292	0.053			
Social Relationships						
Intervention group	13.33 (10.67–13.33)	13.33 (11.33–14.67)	14.67 (12.67–16.00)	0.053	* 0.003	0.068
Control group	13.33 (12.00–14.67)	12.00 (11.33–14.67)	13.33 (12.00–14.67)	0.061	0.849	0.019
*p* (between-group difference)		0.073	0.031			
Environment						
Intervention group	12.50 (11.00–16.00)	14.00 (13.00–15.75)	14.50 (12.75–16.50)	0.126	0.179	0.465
Control group	13.00 (11.25–14.75)	13.00 (11.75–14.50)	13.50 (11.75–15.00)	0.848	0.259	0.417
*p* (between-group difference)		0.102	0.102			

WHOQOL-BREF—WHO Quality of Life Scale Abbreviated Version. * Significant difference. Analysis was adjusted using the Holm–Bonferroni method. Analysis (within-group comparison) was analyzed using a Signed-rank test. Analysis (between-group comparison) was analyzed using a Mann–Whitney U test.

**Table 5:** The themes, categories, and codes (examples) were identified.

Themes	Categories	Codes (Examples)
Increased self-awareness of health and wellbeing	Physiological fitness and health improvement Psychological and emotional balance Social wellbeing and connection Attitudinal change in health concepts	Relaxing physically and feeling comfortable Improving insomnia and sleeping conditions Feeling refreshed and energized for new challenges Relieving stress and anxiety Reinforcing happiness and positive minds Converting negative moods Facilitating connections and relationships with others Strengthening communication skills and self-confidence Clarifying health concerns and beliefs Enhancing intention to help others
Enjoyed diversified and complementary content and activities	Enjoyed in funny laughter yoga exercises Appreciated grateful and happy events daily records Structured and informative mental health knowledge Facilitated flexible participation arrangements	Emphasized body-mind wellness and fitness for good health Enjoyed funny and exciting exercises Reminded appreciation of gratitude and compliments for daily life Increased life satisfaction and meaning Enriched their knowledge, understanding, and strategies for mental problems Supplemented informative and helpful resources Accepted activities arrangement schedules Preferred convenient online or e-learning mode of activities

## Data Availability

The data presented in this study are available on reasonable request from the corresponding author. The data are not publicly available due to ethical restrictions.
